# The Oncogenic EWS-FLI1 Protein Binds *In Vivo* GGAA Microsatellite Sequences with Potential Transcriptional Activation Function

**DOI:** 10.1371/journal.pone.0004932

**Published:** 2009-03-23

**Authors:** Noëlle Guillon, Franck Tirode, Valentina Boeva, Andrei Zynovyev, Emmanuel Barillot, Olivier Delattre

**Affiliations:** 1 Institut Curie, Paris, France; 2 INSERM, U830, Génétique et Biologie des Cancers, Paris, France; 3 INSERM, U900, Cancer et Génome: bioinformatique, biostatistiques et épidémiologie d'un système complexe, Paris, France; Institute of Genetics and Molecular and Cellular Biology, France

## Abstract

The fusion between EWS and ETS family members is a key oncogenic event in Ewing tumors and important EWS-FLI1 target genes have been identified. However, until now, the search for EWS-FLI1 targets has been limited to promoter regions and no genome-wide comprehensive analysis of *in vivo* EWS-FLI1 binding sites has been undertaken. Using a ChIP-Seq approach to investigate EWS-FLI1-bound DNA sequences in two Ewing cell lines, we show that this chimeric transcription factor preferentially binds two types of sequences including consensus ETS motifs and microsatellite sequences. Most bound sites are found outside promoter regions. Microsatellites containing more than 9 GGAA repeats are very significantly enriched in EWS-FLI1 immunoprecipitates. Moreover, in reporter gene experiments, the transcription activation is highly dependent upon the number of repeats that are included in the construct. Importantly, *in vivo* EWS-FLI1-bound microsatellites are significantly associated with EWS-FLI1-driven gene activation. Put together, these results point out the likely contribution of microsatellite elements to long-distance transcription regulation and to oncogenesis.

## Introduction

Ewing tumors, the second most frequent bone tumors in teenagers and young adults, show specific translocations fusing the 5′ part of EWS to the 3′ sequence encoding the DNA binding domain of an ETS factor [1], [2]. In most cases, translocations occur between chromosomes 11 and 22, leading to the formation of the aberrant EWS-FLI1 chimeric transcription factor [3]. In rarer cases, ERG, E1AF, ETV1 or FEV that encode other ETS family members are fused to EWS [4]–[7]. Various experimental procedures, including SELEX experiments and mapping of promoters regulated by EWS-FLI1, have shown that ETS factors bind purine-rich sequences with a GGAA/T core consensus sequence, surrounded by nucleotides that contribute to the specificity of each factor [8]–[11]. This was recently highlighted by a large-scale study of the properties of ETS factors promoter occupancy showing that DNA binding may be divided into two complementary mechanisms [12]. The first would imply a core ETS consensus site that may be recognized by a large proportion of ETS factors, with the consequence of binding of various ETS proteins to common genomic targets. The second process would involve more specific mechanisms, with the recognition of less typical binding sites, possibly in cooperation with other DNA-binding factors.

EWS-FLI1 can recognize *in vitro* the same sequences as FLI-1 [8], but is a more potent transactivator than the wild type factor [13], [14]. It is now largely agreed that EWS-FLI1 oncogenic potential is at least partially mediated by the expression modulation of transcriptional targets. Numerous genes whose expression is modulated by EWS-FLI1 have been described. They exhibit very diverse functions including cell cycle regulation, cell migration, morphogenesis or signal transduction (reviewed in [2]). So far, only few genes have been unambiguously validated as direct EWS-FLI1 targets in the context of Ewing cells. These includes TGFβRII [15], cyclinD1 [16], Id2 and c-Myc [17], IGFBP3 [18], PTPL1 [19], cyclinE [20], MK-STYX [21], caveolin1 [22] and Dax1/NR0B1 [23], [24]. In most cases, one or several ETS consensus sites could be detected in the promoter or first intron of these genes and shown to be crucial for EWS-FLI1 binding and transcription modulation [19], [25]–[28]. EWS-FLI1 may also be associated with other cofactors on particular modular response elements, such as on the Serum Response Element in cooperation with SRF [29], [30], or on composite ETS-AP-1 tandem elements [31].

Recently, two reports indicated that the binding of EWS-FLI1 may not be limited to *bona fide* ETS binding sites but may also occur on GGAA repeats. Indeed EWS-FLI1 regulates the NR0B1 promoter through direct binding to a GGAA microsatellite sequence [32], [33]. Interestingly, a correlation was observed between the number of GGAA modules and the level of NR0B1 expression raising the hypothesis that several EWS-FLI1 monomers may cooperate on a GGAA-rich region [32]. Gangwal et al. conducted a ChIP-chip promoter wide analysis of EWS-FLI1 binding sites and reported that the regulation of other EWS-FLI1 targets may also rely on such microsatellite sequences. So far, the search for EWS-FLI1 targets has been restricted to promoter regions and the precise *in vivo* significance of GGAA microsatellites with respect to expression modulation remains elusive.

In an attempt to decipher a general EWS-FLI1 DNA binding mechanism and to identify candidate direct target genes in the Ewing tumor context, we have combined high throughput sequencing of EWS-FLI1 bound DNA fragments and analysis of EWS-FLI1-induced gene expression modulation. Our approach demonstrates binding of EWS-FLI1 to GGAA-repeat sequences *in vivo* and further shows a binding preference for tracts of 9 repeats or more. We also extend the repertoire of EWS-FLI1 bound GGAA microsatellites and show that, although these sites may be distant from transcription start sites, they are significantly enriched in regions encoding EWS-FLI1 regulated genes. Such results point out the large contribution of GGAA-microsatellite elements to EWS-FLI1 regulation of targets.

## Materials and Methods

### Chromatin immunoprecipitation

Cross-linking was performed with 10^6^ A673, SK-N-MC or MON cells in medium with 1% of formaldehyde for 8 min. Cells were then lysed in 200 µL SDS lysis buffer (1% SDS; 10 mM EDTA; 50 mM Tris, pH 8.1) and sonicated for 10 min at power 3 (20% duty cycles) using ultrasonic processor GE375 apparatus (Meditech Scientific, Clamart, France). Cell lysates were diluted 10 fold in ChIP dilution buffer (0.01% SDS; 1.1% Triton X-100; 1.2 mM EDTA; 16.7 mM Tris, pH 8.1; 167 mM NaCl), precleared for 15 min with protein A-Sepharose and incubated overnight at 4°C with 10 µg anti-FLI-1 C19 antibody (Santa Cruz, CA.). Protein A-Sepharose was then added for 15 min at 4°C. After sequential washes (1× Low Salt Wash Buffer: 0.1% SDS; 1% Triton X-100; 2 mM EDTA; 20 mM Tris-HCl, pH 8.1; 150 mM NaCl; 2× High Salt Wash Buffer: 0.1% SDS; 1% Triton X-100; 2 mM EDTA; 20 mM Tris-HCl, pH 8.1; 500 mM NaCl; 1× LiCl Wash Buffer: 0.25 M LiCl; 1% Igepal; 1% deoxycholic Acid; 1 mM EDTA; 10 mM Tris-HCl pH 8.1; 2× TE Wash Buffer: 10 mM Tris pH 8.1; 1 mM EDTA) and elution from the beads with 1% SDS, cross-links were reversed for 4 h at 65°C. Proteins were then digested by adding 100 µg/mL Glycogen and 200 µg/mL of Proteinase K (Invitrogen, CA) for 1 h at 45°C and DNA, which was recovered by phenol/chloroform extraction, was ethanol precipitated and resuspended in 15 µL of water. DNA was quantified using Quant-iT technology and the Qubit quantification platform from Invitrogen.

### Illumina library construction and sequencing

Immunoprecipitated DNAs were processed and analysed on the Illumina/Solexa platform by the Fasteris company (Geneva, Switzerland). Briefly, DNA ends were repaired using a 1∶5 mixture of T4 and Klenow DNA polymerases following the manufacturer's instructions. After addition of a single adenine base to the DNA using Klenow exo-, adapters were ligated to the ends of the single adenine-tailed purified DNA. Adapter-modified DNA fragments were enriched by PCR using the Phusion polymerase (Finnzymes, Finland) and PCR primer 1.1 and 2.1 (Illumina) following the manufacturer's instructions. DNA was then size-selected at around 300 bp on a 12% PAGE gel. Cluster generation on one channel of the Illumina cell for each sample and 27 cycles of sequencing were performed on the Illumina cluster station and 1G analyzer.

### Processing 1G data

Reads were mapped to the unmasked human reference genome (NCBIv36, hg18) using the Eland alignment tool (Illumina), with a tolerance of up to two mismatches per read sequence. Then, uniquely mapped sequence reads were processed by FindPeaks 3.1.9.2 software [34] in order to detect enriched regions. The threshold of 7 on the minimum peak size was adopted to identify read clusters in EWS-FLI1 cell lines, whereas read clusters in the MON control were selected with a lower threshold of 4. By filtering out clusters common to the Ewing and MON control cell lines, we defined EWS-FLI1 specific areas of enrichment. Since pericentromeric regions are often a source of noise in ChIP-Seq data [35], the corresponding read clusters were removed from subsequent analysis. For enrichment analyses, 50 000 non-overlapping random regions, exclusive of pericentromeric regions, were used as control. These regions were selected to have the same size distribution than the EWS-FLI1-bound regions identified by FindPeaks

### DNA Motif Analyses

ETS binding site analyses were performed using the RegionMiner tool (Genomatix, Germany) with position weight matrices for families of transcription factors or for individual factors. MEME program, version 3.5.1 was used to search for DNA motifs. To generate logos from the MEME output, the WebLogo software program, version 2.8.2 (http://weblogo.berkeley.edu/), was used.

### GGAA microsatellites sequencing

Pairs of primers were designed for each GGAA microsatellite genomic region (listed in Supporting Table S3). After fragment amplification using Phusion polymerase (Finnzymes), DNA was purified with the Nucleofast system (Macherey-Nagel, Hoerdt, France) and sequenced using Big Dye V1.1 (Applied Biosystems, Courtaboeuf, France).

### Luciferase assays

Varying numbers of GGAA motifs were cloned in the pGL3-promoter vector (Promega, Charbonnieres, France). EWS-FLI1 cDNA was cloned in the pCDH1-MCS1-puro vector (System Biosciences, CA). 293T and shA673-1C cells were transfected with firefly reporters, the renilla encoding plasmid (pREP7-Rluc, kindly provided by Keji Zhao) and pCDH1-EWS-FLI1 or control plasmids. Firefly activity was normalized to Renilla luciferase activity to adjust differences in transfection efficiency.

## Results

### EWS-FLI1 binds *in vivo* to GGAA microsatellites and GGAA-rich sequences

We used chromatin-immunoprecipitation coupled to high throughput sequencing (ChIP-Seq) to construct a high-resolution EWS-FLI1-binding map. Immunoprecipitation experiments were conducted in SK-N-MC and A673, two Ewing cell lines that express type 1 EWS-FLI1, and in MON, a malignant rhabdoid tumor (MRT) cell line. The antibody that was used is directed against the C-terminus part of FLI1. It could theoretically immunoprecipitate wild type FLI1, however this protein is expressed in none of the three afore-mentionned cell lines. We choose the MON cell line as a control because Ewing and MRTs share common characteristics: they both belong to the group of small round cell tumors of children and may share a mesenchymal stem cell of origin [36], [37]. However, MRTs do not harbor the EWS-FLI1 rearrangement.

For each sample, between 1.9 and 3.5 million sequences with a mean length of 35 nt were obtained. Of these, approximately 80% had a single location on the human genome (Table 1). Analysis of these sequences was carried out with the FindPeaks program [34]. This identified 26, 94 and 195 EWS-FLI1 specific read clusters in the SK-N-MC and in each of the two A673 cell line samples, respectively. Read clusters were selected as EWS-FLI1 specific if no cluster was found at the same position in the MON control. A total of 246 regions was thus identified as EWS-FLI1 specific (Table S1), 14 being specific to SK-N-MC cell line, 220 to A673 and 12 common to both cell lines. The size of identified regions varied from 329 to 2247 bp with an average length of 725 bp.

**Table 1:** Number of reads and corresponding mapped sequences per Chip-Seq experiments.

Reads	SK-N-MC	A673 (1)	A673 (2)	MON (control)
Total sequenced	2,961,880	1,888,878	3,466,371	2,473,927
Total uniquely mapped	2,577,613	1,656,023	3,004,601	1,982,019

**Figure 1:**
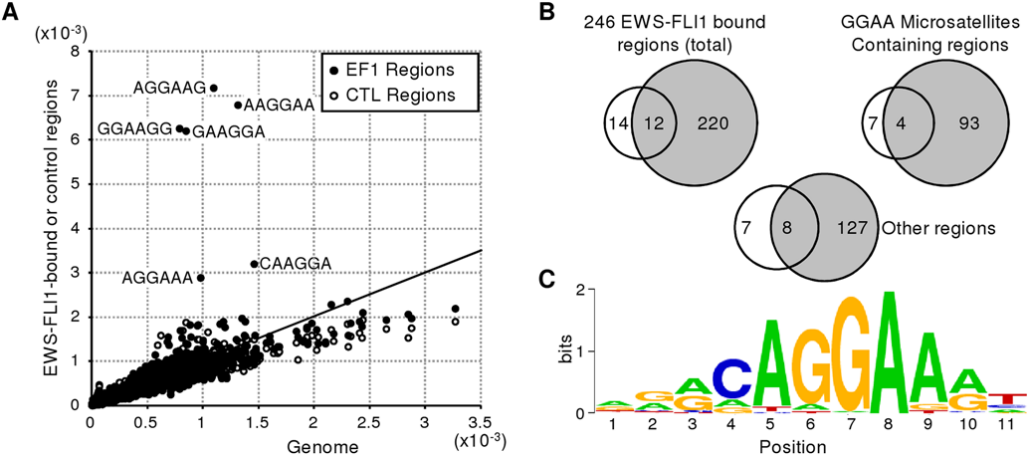
EWS-FLI1 binds GGAA microsatellites or GGAA-rich sequences. A. Enrichment of GGAA motifs in EWS-FLI1-bound sequences. Frequencies of each of 4096 possible 6mer nucleotides found for the 246 identified EWS-FLI1 specific regions (black circle) and for regions identified in the control experiment (white circle) are represented along the Y axis whereas frequency of the same 6mers in the genome is represented on the X axis. B. GGAA repeat enrichment is a common feature of Ewing cell lines. Number of sequences found in A673 (grey circle) and SK-N-MC (white circle) for each type of binding site. C. Consensus motif assessed with MEME algorithm (E-value = 4.1×10^−46^) in regions other than GGAA microsatellites.

In order to characterize EWS-FLI1 consensus binding sites, over-representation of sequence motifs was searched for. Frequencies of every possible 4–8 bp long oligomer were assessed in the 246 EWS-FLI1 specific regions compared to their respective frequencies in the human genome. A clear over-representation of oligomers containing GGAA motifs was observed (results obtained for 6-mer motifs are displayed in Fig. 1A). More precisely, 104 regions presented microsatellite sequences consisting of 3 or more GGAA-containing tandem repeats: (GGAA)_n_, (GGAAN)_n_ or (GGAANN)_n_. The other 142 regions did not contain such microsatellites. Both types of regions were found in A673 and SK-N-MC cell lines (Fig. 1B), indicating that neither type of region was cell specific. The RegionMiner and MatInspector softwares (Genomatix) were used to assess whether the two types of EWS-FLI1 specific regions were enriched in *bona fide* ETS factor binding sites. Regions containing microsatellites did not show any additional ETS consensus over-representation after repeat filtration (Table S2). In contrast, a clear over-representation of ETS family binding motifs was observed in the EWS-FLI1-specific regions that do not contain microsatellite sequences (Table 2). These regions also presented very frequent combination of two ETS sites or of ETS site with consensus sites for other transcription factors (Table 3). These non-microsatellite EWS-FLI1 specific regions were also analyzed with the MEME software that defines position weight matrices giving frequency distributions of each base at each position [38]. As shown in Figure 1C, MEME retrieved a consensus sequence highly similar to an ETS binding sequence.

**Table 2:** Transcription factor consensus sites enrichment in regions other than GGAA microsatellites.

TF Matrices	Over representation (1)	Z-Score (1)	Number of Matches
V$ELK1.02	10.4	41.82	207
V$CETS1P54.01	6.83	35.63	256
V$ETS1.01	5.76	29.28	219
V$ETS2.01	4.14	26.97	306
V$ELK1.01	5.62	26.63	188
V$FLI.01	5.86	26.39	174
V$ELF2.01	4.31	24.5	237

(1) Compared to the genomic representation.

**Table 3:** Transcription factor modules containing an ETSF binding site in regions other than GGAA microsatellites.

Modules with V$ETSF	Over representation (1)	Z-Score (1)	Number of Matches
V$ETSF-V$ETSF	5.43	41.08	468
V$ETSF-V$GREF	5.22	30.55	275
V$ETSF-V$HOXF	2.18	17.92	504
V$CREB-V$ETSF	2.74	16.68	254
V$ETSF-V$NKXH	2.18	14.67	338
V$ETSF-V$NFKB	3.5	14.26	115
V$AP4R-V$ETSF	4.98	13.32	57
V$ETSF-V$NOLF	4.13	13.03	73
V$E2FF-V$ETSF	2.94	12.54	124
V$ETSF-V$OCT1	2.09	12.5	277
V$ETSF-V$ZBPF	2.78	12.48	138
V$ETSF-V$PAX1	6.85	12.42	32
V$ETSF-V$HAND	2.5	12.17	166
V$ETSF-V$NR2F	2.23	11.79	206
V$ETSF-V$MOKF	3.22	11.72	91
V$ETSF-V$SORY	1.95	11.08	265
V$ETSF-V$PARF	2.05	10.98	227
V$ETSF-V$HEAT	2.37	10.81	149
V$ETSF-V$MEF3	6.01	10.78	29
V$ETSF-V$PTF1	4.02	10.17	47
V$BTBF-V$ETSF	4.52	10.03	38

(1) Compared to the genomic representation.

These observations suggested at GGAA microsatellites and *bona fide* ETS containing regions constitute two types of EWS-FLI1 binding regions in Ewing cells.

### EWS-FLI1 preferentially binds microsatellites with more than 9 GGAA repeats

**Figure 2:**
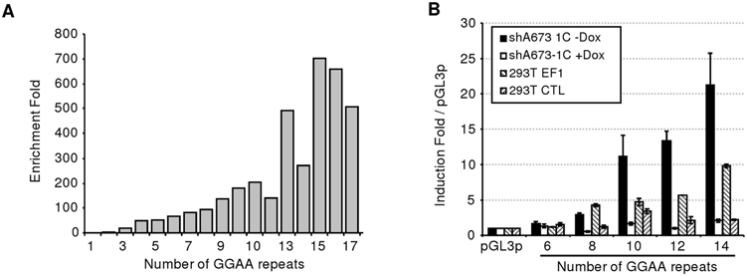
EWS-FLI1 microsatellite length preferences. A. Ratio of the number of GGAA repeats in EWS-FLI1-bound regions to the number of repeat in 50000 randomly picked regions. B. Ability of EWS-FLI1 to modulate transcription of a reporter gene depending upon the number of GGAA repeats. Firefly relative to Renilla luciferase activity is shown. Control experiments with the empty pGL3-promoter vector were set to 1.

In order to analyze whether EWS-FLI1-binding was skewed toward particular numbers of GGAA repeats we compared the number of GGAA repeats between EWS-FLI1-bound and random regions. The mean number of GGAA amongst the 246 EWS-FLI1-bound regions over the mean number of GGAA amongst random regions was dramatically increased. This was particularly obvious for a number of GGAA higher than 9 (Fig. 2A). In order to evaluate the size of the microsatellites in Ewing cells, the sequence of 51 EWS-FLI1-bound microsatellites was determined in the A673 and SK-N-MC cell lines. This showed that most microsatellites were polymorphic. However, the range of GGAA repeats number was consistent with that reported in public database (Table S1). Altogether, these data suggest that EWS-FLI1 may preferentially bind *in vivo* microsatellites with more than 9 repeats (hereafter called microsatellites>9R).

To test the responsiveness of such microsatellites structures to EWS-FLI1, luciferase assays were performed using different numbers of GGAA repeats cloned into the pGL3-promoter reporter vector (Fig. 2B). Experiments were performed in a Ewing cell line that contains a doxycyclin-regulated *EWS-FLI1* specific shRNA, shA673-1C [37], and in 293T cells transfected with an *EWS-FLI1*-expression vector. In both cases, in the presence of EWS-FLI1, very strong luciferase activities could be detected with the constructs containing at least 10 GGAA repeats while mild luciferase activities were detected when the constructs contained a lower number of repeats. These luciferase activities were dependent on EWS-FLI1 since doxycyclin inhibition of EWS-FLI1 expression in shA673-1C (+Dox) or transfection of 293T with empty vector (293T CTL) led to little or no activation of the reporter gene (Fig. 2B).

### Enrichment for EWS-FLI1 regulated genes around binding sites

**Figure 3:**
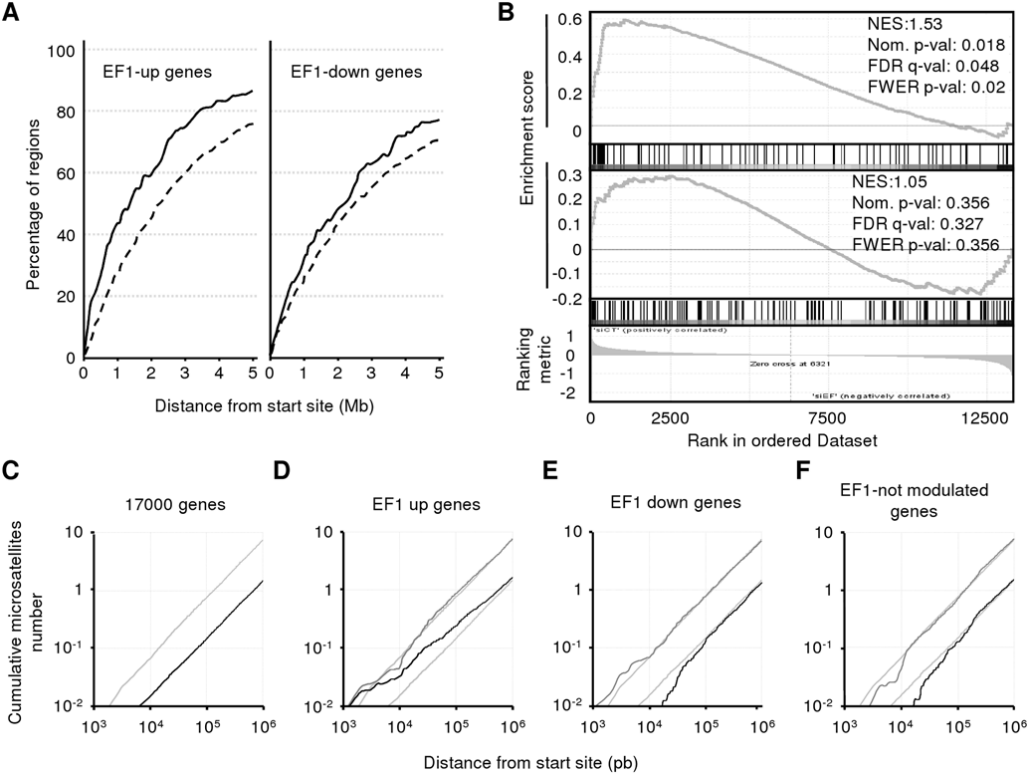
Long distance EWS-FLI1 binding on GGAA microsatellites results in significant gene expression activation. A. Proportion of EWS-FLI1-bound regions, as compared to the proportion of random regions, around EWS-FLI1 regulated genes. The proportion of EWS-FLI1-bound regions as a function of the distance to the transcription start sites of EWS-FLI1-up or -down regulated genes (solid lines) is shown. As a control, a similar function is indicated for 1500 randomly chosen regions (dashed line). B. Gene Set Enrichment Analysis (GSEA) of genes flanking EWS-FLI1-bound microsatellites. The 94 genes flanking the 80 microsatellites>9R regions (upper panel) as well as the 144 genes flanking the non-microsatellites regions (lower panel) were used as gene set. The expression dataset resulted from previously described EWS-FLI1 inhibition experiments of A673 and SK-N-MC Ewing cell lines [37], [40], ranked using the signal-to-noise algorithm. A strong enrichment of genes flanking EWS-FLI1 bound GGAA microsatellites among EWS-FLI1 up-regulated genes is observed (upper panel). C–F. Regions upstream of EWS-FLI1 up-regulated genes are enriched in GGAA-microsatellites. The number of microsatellites with either 3 to 9 GGAA repeats (grey line) or more than 9 repeats (black line) was calculated for each 1 Kb window from 1 Kb to 1 Mb upstream of the transcription start sites. The numbers of GGAA repeats along DNA are shown for (C) 17000 known genes (control distribution), (D) 582 EWS-FLI1-up-regulated genes, (E) 558 EWS-FLI1-down-regulated genes and (F) 561 genes that are expressed in A673 and SK-N-MC cell lines but not regulated by EWS-FLI1. The control distribution shown in C is also indicated on part D, E and F.

Among the 246 EWS-FLI1 specific regions, 146 were localized in intergenic regions, 13 in exons, 79 in gene introns and 8 in promoters. These EWS-FLI1 binding sites were very frequently located far away from any transcription unit, with a mean distance to transcription start sites of 242 Kb and up to 3 Mb. To address the issue of a potential link between EWS-FLI1 bound regions and EWS-FLI1 regulated transcription, we compared the distances of the 246 EWS-FLI1-specific regions or of randomly picked regions to the nearest EWS-FLI1 regulated gene. We used a previously published list of EWS- FLI1 regulated genes that were identified through shRNA inhibition experiments in A673 and SK-N-MC Ewing cell lines [37]. This list contains 557 and 577 genes that are down- or up-regulated by EWS-FLI1, respectively (fold change>|2| with a Welsh p-value<0.01). Figure 3A shows the percentage of EWS-FLI1-bound or random regions with an EWS-FLI1-modulated gene at a given distance. It is interesting to note that about 43% of the 246 EWS-FLI1 bound regions have the transcription start site of an EWS-FLI1-up-regulated gene within 1 Mb (as compared to 27% for random regions) and 60% within 2 Mb (46% for random). The increased proportion of EWS-FLI1-down-regulated genes located within 1 or 2 Mb of EWS-FLI1 regions is less obvious (31% as compared to 24% for random regions and 47% as compared to 42%, respectively). These results indicated that the 246 EWS-FLI1 bound regions were significantly closer to EWS-FLI1-regulated genes than randomly selected regions (Mann-Whitney p-value<10^−16^). However, no correlation between expression level of genes and their distance to microsatellites>9R could be found. To further analyze the link between EWS-FLI1 transcriptional expression modulation and EWS-FLI1-bound microsatellites, GSEA analyses were performed [39]. As expression dataset, we used the afore-mentioned published data [37], [40], ranked using the signal-to-noise metric. The gene set contained the genes flanking the 80 regions containing the microsatellites>9R. As shown on the upper panel of Figure 3B, the gene set is overrepresented at the left edge that contains EWS-FLI1 up-regulated genes. Indeed, among the 94 genes flanking the microsatellites>9R, 30 were at the leading edge (Z-score = 8.6, Fisher p-value = 2.1×10^−11^). GSEA analysis carried on the regions bound by EWS-FLI1 that do not contain GGAA microsatellite is shown on Figure 3B, lower panel. This shows that relative enrichments are observed at both edges, however the GSEA overall statistics do not reach significance. This analysis demonstrated that EWS-FLI1 up-regulated genes are significantly enriched in the vicinity of EWS-FLI1-bound microsatellites with more than 9 GGAA repeats therefore suggesting that microsatellites>9R are associated with a function of EWS-FLI1 in transcription activation.

Reciprocally, we investigated whether upstream regions of EWS-FLI1 modulated genes were enriched with microsatellites>9R. The 1 Kb cumulative frequency of GGAA repeats was calculated from the transcription start site to 1 Mb upstream of EWS-FLI1-regulated genes [37], as well as of a set of 561 control genes that were found expressed but not modulated in the same experiments (Fold Change<|1.1| with a log2 expression value between 4 and 7). These frequencies were then compared to the frequency of GGAA repeats found up to 1 Mb upstream of the start sites of 17000 known genes (Fig. 3C). The number of GGAA microsatellites>9R located upstream of EWS-FLI1-up-regulated genes was clearly higher than for other known genes (Fig. 3D, Mann-Whitney test p-value<10^−12^). This overrepresentation was observed neither for small (3 to 9 repeats) microsatellites nor in the upstream regions of EWS-FLI1-down-regulated genes (Fig. 3E) nor for genes that are expressed in Ewing cells but not modulated by EWS-FLI1 (Fig. 3F). Moreover, the same enriched distribution was not observed for GGAT repetitions (data not shown). This *in silico* analysis shows that upstream regions of EWS-FLI1 up-regulated genes are enriched for GGAA microsatellites.

Overall, these observations strongly suggest that a large part of EWS-FLI1 DNA binding is driven by GGAA sequence recognition and correlates with genes expression activation through EWS-FLI1 driven long-distance control of transcription.

## Discussion

EWS-FLI1 driven oncogenesis is thought to rely mainly on DNA binding and subsequent alteration of the expression of specific target genes. Up to now, studies aiming at finding EWS-FLI1 target genes investigated exclusively binding to promoter regions either through genome wide approaches or through specific analyses of genes transcriptionally modulated by this oncogene. In order to identify EWS-FLI1 specific *in vivo* target genes in an unbiased genome wide approach, we used here chromatin immunoprecipitation coupled with high throughput sequencing.

Our findings uncover two types of EWS-FLI1 binding sequences: (i) consensus ETS binding sites and (ii) GGAA microsatellites. The former correspond to the binding sites that are expected for the EWS-FLI1 factor, considering its common binding properties with wild type FLI1. Our approach not only broadens the list of such sites as EWS-FLI1 direct targets, but also points out their significant association in pairs or with other transcription factors binding sites within modules. The association of ETS binding sites with binding sites for factors such as CREB or NFkB may suggest a cooperative interplay of EWS-FLI1 with other cancer-related factors. The present identification of GGAA microsatellites as EWS-FLI1 targets confirms and extends a previous ChIP-on-chip-based, genome-wide analysis of EWS-FLI1 binding sites in promoter regions. Indeed, GGAA microsatellites were recently described as EWS-FLI1 binding sites within different promoters, including NROB1, FCGRT and caveolin 1. Moreover, EWS-FLI1 direct interaction with these repeated elements was validated by gel shift assays [33].

The aforementioned publication describing microsatellites as EWS-FLI1 targets pointed out a requirement for minimal length of four GGAA repeats for binding. Our study further indicates that a strong *in vivo* overrepresentation is observed for microsatellites containing between 9 and 17 repeats. In agreement with the hypothesis that such repeats play a role in EWS-FLI1-driven transcription regulation, we observe that a dramatic effect on expression of a reporter gene is indeed observed for this range of repeats both in heterologous 293T and Ewing cells. This is also in agreement with a recent study on NR0B1 showing that the level of expression of this gene in different Ewing cell lines is correlated to the number of GGAA repeats in its promoter [32]. Yet, the precise mechanism underlying such binding needs further investigation. Cooperative binding or increased probability of binding due do the high local concentration of binding sites have been proposed [32], [33]. The DNA conformation, and in particular the DNA bending that has been previously shown to be crucial for ETS factors' binding, may also be influenced by the number of GGAA repeats [41]–[43]. Further ChIP-Seq experiments are required to increase the depth of the analysis and evaluate *in vivo* the potential of EWS-FLI1 to bind different microsatellite sequences. In particular, this will enable to search for the presence in the vicinity of GGAA repeats of binding sites for specific transcription factors that may cooperate with EWS-FLI1 for binding. It will also be very informative to combine these EWS-FLI1 analyses with genome-wide studies of epigenetic landmarks since chromatin conformation may be crucial for EWS-FLI1 binding.

Combining the ChIP strategy to global gene expression microarrays reveals that sites with long GGAA microsatellites are preferentially localized near EWS-FLI1 positively modulated genes. Several EWS-FLI1 modulated genes located in the vicinity of GGAA repeats can now be tested for their implication in Ewing sarcoma oncogenesis, such as the kinases DLG2 and VRK1, the latter being involved in cell cycle regulation possibly through the regulation of p53 function [44], [45]. Interestingly, EWS-FLI1 gene modulation *via* microsatellites targeting might be more general than suggested by the present analysis as a number of EWS-FLI1 up-regulated genes that present long GGAA microsatellite sequences within 1 Mb of their transcription start sites are not detected here. In particular, the previously described NR0B1 promoter locus is not retrieved with the criteria that were used. However, it is noteworthy that two independent reads were found at the expected location in the A673 cell line. Nevertheless, other genes, like TGFBR2, known to be targeted by EWS-FLI1 were not recovered in our experiments. Moreover, we observed a relatively poor overlap of the sites found in the two Ewing cell lines. Taken together, these observations indicate that a total of 3 million reads per sample is obviously not sufficient for a saturating genomic coverage. More reads are certainly required for an in depth study of transcription factors such as EWS-FLI1.

Amongst the 80 microsatellites>9R bound by EWS-FLI1 only 5 were found within the first 10 kb upstream of genes (see Table S1) amid which 4 were found to be regulated by EWS-FLI1 in our experiments (CAV1, FCGRT, FVT1/KDSR and ABHD6). To address more globally the question of the putative correlation between position and expression level, we studied the mean distances of GGAA microsatellites>9R to genes located at the leading edge in the GSEA analysis as compared to the other genes in the same geneset. Although, we observe a trend toward a shorter distance (267276 bp+/−356993 bp versus 494046 bp+/−675168 bp) it does not reach significance (welsh p-value = 0.09). Therefore, the bias that we observe for short distances is less obvious that the one described in a recent report [33]. Indeed, we observed a significant enrichment of microsatellites>9R in the first 5 kb upstream of up-regulated genes but they only accounted for 1.5% of the microsatellites>9R found within 1 Mb upstream of up-regulated genes. This relative discrepancy between both studies may probably be explained by the distinct statistical methods that were applied. Gangwal *et al.* performed a statistical analysis at each individual ranked position whereas we estimated the significance of the overall distribution of the GGAA microsatellites with respect to the distance to start sites of EWS-FLI1 regulated genes. In such an analysis, even when the GGAA microsatellites located at less than 5 kb are removed, the analysis remains highly significant indicating that the effects of GGAA microsatellites may not be limited to the first 5 kb upstream of the genes. An important finding of this work is thus that most EWS-FLI1 binding sites appear to be localized quite far from gene transcription start sites. This indicates that EWS-FLI1 does not bind and act exclusively through promoter regions but can also impact transcription at long distance. Such long distance expression control has been described for several transcription factors in locus control regions, epitomized by the β-globin locus (for review, see [46]). Moreover, computational prediction of transcriptional regulatory modules also revealed putative position of transcription factor binding sites far away from coding sequences [47] and gene deserts are now scanned in search for enhancer modules [48]. In addition, very distant genomic region looping has been demonstrated to promote transcription in transcriptional hubs (reviewed in [49], [50]). Future analyses by chromosome conformation capture of long range interactions between EWS-FLI1 binding sites, and in particular GGAA repeats, with other loci are required to study the nuclear architecture of EWS-FLI1 bound domains.

Finally, it is noteworthy that microsatellite sequences have previously been associated with genes regulation. Indeed, long tandem repeats of CCGCC sequence in the promoter of the *SMYD3* histone methyltransferase have been linked to an increased binding and transactivation by E2F-1 [51]. Moreover, in this last study, the allele corresponding to the longest CCGCC repeat was shown to be more represented in individuals with colorectal cancer, hepatocellular cancer or breast cancer, thus suggesting a possible role in cancer susceptibility. Polymorphisms in GGAA repeat numbers of key EWS-FLI1 targets may similarly constitute attractive candidates to account for Ewing sarcoma susceptibility [52].

## Supporting Information

Table S1 246 EWS-FLI1-bound regions description
(0.81 MB XLS)

Table S2 Transcription factor consensus sites enrichment in regions containing GGAA microsatellites, after filtration of the GGAA repeats
(0.03 MB DOC)

Table S3 Oligonucleotides used for microsatellite sequencing
(0.03 MB XLS)
