# Expression of Concern: The Oncogenic EWS-FLI1 Protein Binds *In Vivo* GGAA Microsatellite Sequences with Potential Transcriptional Activation Function

**DOI:** 10.1371/journal.pone.0270655

**Published:** 2022-06-23

**Authors:** The PLOS ONE Editors

After this article [1] was published, concerns were raised about the availability of the ChIP-sequencing data and microsatellite sequencing data in this article. The *PLOS ONE* data availability policy [2], applicable at the time the article was submitted to the journal, requires that authors must comply with best practice in their discipline at the time, specifically the deposition of sequencing data in an appropriate public database.

Upon follow up with the authors, the sequencing data has not been provided. In light of this, the *PLOS ONE* editors are issuing this Expression of Concern to make readers aware about the unavailability of sequencing data related to [1].
