# Supplementary material for: The Oncogenic EWS-FLI1 Protein Binds In Vivo GGAA Microsatellite Sequences with Potential Transcriptional Activation Function
Source: PLoS One. 2009 Mar 23;4(3):e4932. doi: 10.1371/journal.pone.0004932 (PMC2654724; doi:10.1371/journal.pone.0004932)
Supplement: Table S2 — Transcription factor consensus sites enrichment in regions containing GGAA microsatellites, after filtration of the GGAA repeats (0.03 MB DOC) [file pone.0004932.s002.doc]

| **Cell Line** | **Modules with V$ETSF** | **Number of matches** | **Over representation (1)** | **Z-Score (1)** |
| --- | --- | --- | --- | --- |
| A673 (27) | V$ETSF-V$HOXF | 101 | 1.91 | 6.55 |
|  | V$ETSF-V$TBPF | 50 | 1.59 | 3.23 |
|  | V$ETSF-V$ETSF | 38 | 1.93 | 4 |
| SK-N-MC (11) | V$AP4R-V$ETSF | 13 | 3.04 | 3.98 |
|  | V$ETSF-V$SMAD | 10 | 7.14 | 6.84 |
|  | V$ETSF-V$TEAF | 8 | 4.02 | 3.9 |

**Supplemental Table S2.** Transcription factor consensus sites enrichment in regions containing GGAA microsatellites, after filtration of the GGAA repeats

(1) Compared to the genomic representation
